# Glucose Deprivation Induced by Acarbose and Oncolytic Newcastle Disease Virus Promote Metabolic Oxidative Stress and Cell Death in a Breast Cancer Model

**DOI:** 10.3389/fmolb.2022.816510

**Published:** 2022-07-22

**Authors:** Qayssar A. Obaid, Ahmed Majeed Al-Shammari, Khalisa K. Khudair

**Affiliations:** ^1^ Department of Animal Production, College of Agriculture, University of Sumer, Dhi Qar, Iraq; ^2^ Department of Experimental Therapy, Iraqi Centre for Cancer and Medical Genetic Research, Mustansiriyah University, Baghdad, Iraq; ^3^ Department of Physiology and Pharmacology, College of Veterinary Medicine/Baghdad University, Baghdad, Iraq

**Keywords:** glucose deprivation, oncolytic virotherapy, oxidative stress, apoptosis, cancer

## Abstract

Cancer cells are distinguished by enhanced glucose uptake and an aerobic glycolysis pathway in which its products support metabolic demands for cancer cell growth and proliferation. Inhibition of aerobic glycolysis is a smart therapeutic approach to target the progression of the cancer cell. We employed acarbose (ACA), a particular alpha-glucosidase inhibitor, to induce glucose deprivation combined with oncolytic Newcastle disease virus (NDV) to enhance antitumor activity. In this work, we used a mouse model of breast cancer with mammary adenocarcinoma tumor cells (AN3) that were treated with ACA, NDV, and a combination of both. The study included antitumor efficacy, relative body weight, glucose level, hexokinase (HK-1) level by ELISA, glycolysis product (pyruvate), total ATP, oxidative stress (ROS and reduced glutathione), and apoptosis by immunohistochemistry. The results showed significant antitumor efficacy against breast cancer after treatment with combination therapy. Antitumor efficacy was accompanied by a reduction in body weight and glucose level, HK-1 downregulation, inhibition of glycolysis products (pyruvate), total ATP, induction of oxidative stress (increase ROS and decrease reduced glutathione), and apoptotic cell death. The findings propose a novel anti–breast cancer combination involving the suppression of glycolysis, glucose deprivation, oxidative stress, and apoptosis, which can be translated clinically.

## Introduction

Even in the presence of oxygen, breast cancer cells rely on fermentative aerobic glycolysis rather than oxidative phosphorylation, which requires huge amounts of glucose to create energy and support metabolic function (i.e., the Warburg effect) (Zheng, 2012). Cancer cells grow rapidly compared to normal cells, requiring an increase in ATP to meet their metabolic demands (Romero-Garcia et al., 2011). Cancer cells (in comparison to normal cells) exhibit signs of oxidative stress. Aerobic glycolysis benefits cancer cells by generating fewer reactive oxygen species (ROS). (Cairns et al., 2011). Thus, upregulation of glycolysis may be an adaptive response of cancer cells to increase ATP production in an oxygen-deprived environment and promote mitochondrial resistance to pro-apoptotic permeabilization. Pro-apoptotic factors (e.g., ions, proteins, ROS) are downregulated, while antiapoptotic factors (e.g., Bcl-2, ANT2, and chaperones) and antioxidant enzymes are upregulated (Indran et al., 2011). When ROS levels are exceedingly high, p53 launches a failsafe apoptosis program, boosting ROS levels *via* the mitochondrial apoptosis pathway to assure cell death (Liu et al., 2008). Numerous cancer therapies are based on inhibiting this metabolic pathway. It is well-established that glucose deprivation has a detrimental effect on cancer glycolysis and may even result in cell death (Gatenby and Gillies, 2007). Withdrawal of glucose initiates a positive feedback loop in which NADPH oxidase and mitochondria generate reactive oxygen species (ROS), and protein tyrosine phosphatases are inhibited by oxidation and ROS-mediated cell death (Graham et al., 2012).

Acarbose is a glucoregulatory drug; it induces glucose deprivation *via* competitive alpha-amylase and alpha-glucosidase inhibitors, retards the digestion of complex dietary carbohydrates in the small intestine’s brush border, and reduces the rapid rise in blood glucose following a meal (postprandial) (Chiasson et al., 2003). Acarbose is safe and well-tolerated, with a low incidence of adverse effects. Acarbose recipients’ most common adverse events were gastrointestinal (abdominal pain, flatulence, and diarrhea) (Neuser et al., 2005; He et al., 2014). Besides its role as an FDA-approved medication for type II diabetes and hyperglycemia, acarbose has been explored as a calorie restriction mimetic (CRM) in longevity/healthy aging studies (Gibbs et al., 2018). CRMs are agents that mimic the benefits of caloric restriction (e.g., increased longevity and delayed onset of age-related illnesses) without restricting calorie consumption. To substantiate this, acarbose was found to prolong the lifespan of mice (Harrison et al., 2019) and is associated with a dose-dependent decline in the frequency of colon cancer in type II diabetic patients (Tseng et al., 2015).

Virotherapy is an intelligently targeted therapy since it enters and eliminates cancer cells while sparing healthy tissue. Oncolytic virotherapy using NDV was found safe even with extremely high doses in experimental animals and humans in clinical trials (Freeman et al., 2006; Schirrmacher, 2016; Ali et al., 2021; Al-Shammari et al., 2022). The viral dose injected into the tumor mass multiplies and is replicated unless eliminated by the immune system (Kirn et al., 2001; Al-Shammari et al., 2014a). Using virotherapy alone has thus far been unable to eradicate malignancies in animal and clinical trials. The most effective approach to completely eradicate the tumor is combining oncolytic virus therapies with other treatment options like gene therapy and radiation/chemotherapy (Chu et al., 2004). Oncolytic virus-based cancer virotherapy has been shown to be successful when combined with chemotherapies and radiotherapy (Harrington et al., 2010; Al-Shammari et al., 2016). Although malignant tumors are generally incurable diseases, oncolytic virotherapy research (for treatment with viruses that infect and kill cancer cells) develops quickly (Ottolino-Perry et al., 2010). Combination therapy aims to attack tumor cells *via* multiple approaches to prevent cancer cells from acquiring resistance to treatment (Kumar et al., 2008). NDV has been used to treat breast cancer by inhibiting glycolysis and downregulating GAPDH and hexokinase-2 (Al-Shammari et al., 2019; Al-Ziaydi et al., 2020a). The present study aims to investigate using acarbose, a glucosidase inhibitor, to induce glucose deprivation to increase breast cancer cell sensitivity to oncolytic NDV and understand the mechanisms of the combination therapy that enhances cell death.

## Materials and Methods

### The Virus

The Iraqi attenuated NDV strain (Iraq/Najaf/ICCMGR/2013) named AMHA1 (Al-Shammari et al., 2014b) was provided by the Cell Bank Unit, Experimental Therapy Department, Iraqi Center of Cancer and Medical Genetics Research (ICCMGR), Mustansiriyah University. The Iraqi AMHA1 strain was propagated in the embryonated chicken eggs (Al-Kindi Company, Baghdad, Iraq), harvested from allantoic fluid, and then purified from debris through centrifugation (3,000 rpm, 30 min at 4°C). NDV was quantified through a hemagglutination test, aliquoted, and stored at −80°C. Viral titers were determined based on 50% tissue culture infective dose titration on Vero cells following the standard procedure (Hindi et al., 2017).

### Animals

Swiss Albino female mice were housed under the ICCMGR protocols. The scientific committee of Baghdad University, College of Veterinary Medicine, authorized all experimental protocols, including ethical approval (2651/DA in 17/12/2019 from the College of Veterinary Medicine/University of Baghdad).

### Experimental Design

This experiment utilizes the breast cancer model, the mammary adenocarcinoma tumor known as AN3 (Al-Shammari et al., 2008). The tumor was allotransplanted in inbreed mice, which allowed for its continuous propagation in mice. Tumors were established by injecting AN3 cells (1×10^6^/100 μl per site) into the right flanks of female Swiss Albino mice aged 6–8 weeks. When the tumor nodules attained a diameter of 0.5–1 cm, the animals were separated into four groups of 10 randomly: First group: mice in this group were injected with i/p of 0.9% normal saline and received a normal diet (control group); second group: mice in this group received acarbose 1,000 ppm with diets daily (Harrison et al., 2019); third group: mice in this group were injected with NDV 70,000000 intratumorally in a single dose (Al-Shammari et al., 2019); and fourth group: mice in this group received acarbose 1,000 ppm with diets daily for 18 days plus NDV 70,000000 intratumorally in a single dose. After 18 days, the mice were anesthetized and killed with a fatal dose of chloroform. *In vivo* experiments were repeated twice.

### Evaluation of Antitumor Efficacy

The tumor diameters were measured every third day, and their sizes were determined using calipers. The tumor volume was determined as the mean SD for each group using the formula (product of 0.5 length breadth width) (Al-Shammari et al., 2011). To calculate tumor growth, the tumor volume was standardized to the volume of each tumor at time zero, corresponding to the start of therapy. During the evaluation period, tumor growth inhibition (TGI) was determined twice weekly using the following formula (Phuangsab et al., 2001):

GI% = tumor volume in the untreated group—tumor volume in the treated group/tumor volume in the untreated group x100 (1).

A tumor growth inhibition of greater than 50% was regarded as significant.

### Relative Body Weight

The bodyweight of each mouse was weighed every third day using a sensitive balance. The relative body weight was calculated as RBW = (bodyweight on a measured day)/(bodyweight on day 0) × 100.

### Glucose Levels

Blood glucose concentrations were quantified using a glucometer and test strips (Contour, Japan). The blood sample was obtained from the tail vein during the amputation of the tail.

### Hexokinase-1 Enzyme Quantification

To determine the concentration of the hexokinase enzyme, the mouse mammary adenocarcinoma tissue sample was weighed and homogenized in PBS on ice, followed by 5-min centrifugation at 5000 g to get the supernatant. The Hexokinase enzyme concentration was measured using a quantitative ELISA kit (Elabscience, United States) following the manufacturer’s instructions for a Hexokinase-1 assay.

### Pyruvate Assay

The pyruvate concentration was measured using a colorimetric assay with a pyruvate assay kit (Elabscience, United States). On ice, a weighted mouse mammary adenocarcinoma tissue sample was homogenized in normal saline. To extract the supernatant, the tissue homogenate was centrifuged for 10 min at 3,500 g.

### ATP Assay

ATP contents were determined using a colorimetric method using an ATP assay kit (Elabscience, United States). A fresh mouse mammary adenocarcinoma tissue sample was weighed and cut into pieces, then added to boiled distilled water and incubated in a boiling water bath for 10 min, and then mixed fully for 1 min. The samples were centrifuged for 10 min at x10000 g. The supernatant was collected for measurement (according to the manufacturer’s instructions). The principle of the detection kit is that creatine kinase catalyzes creatine and adenosine triphosphate to generate creatine phosphate, then identified by phosphomolybdic acid colorimetry.

### Reactive Oxygen Species Assay

ROS was measured through a fluorometric method by using the ROS assay kit (Elabscience, United States). The level of intracellular ROS was monitored in the mouse mammary adenocarcinoma tissue in the treated and control animals.

The recommended protocol was started by preparing a single-cell suspension using enzymatic digestion. This is carried out by immediately taking the mouse mammary adenocarcinoma tissue into a precooled Reagent 3 working solution and cleaning the blood and other contaminants from the tissue. The massive compositions like fiber, fat, and blood vessels were removed. The remaining tissue was minced into about 1 mm3 piece with ophthalmic scissors; then, we immersed these pieces in precooled Reagent 3 working solution to remove the cell debris. Then, we added an appropriate amount of digestion enzyme and incubated it in a 37°C water bath for 20–30 min. To stop the digestion, we added Reagent 3 working solution. After that, we filtered the mixture to remove the massive tissue component using nylon mesh (300 mesh) and collected only the cells. The cell suspension was centrifuged at 500 g for 10 min, and the supernatant was discarded; then, the cell pellet was washed with Reagent 3 working solution two times. Finally, cells were resuspended to prepare the single-cell suspension solution. The cell amount was about 10^6^.

The fluorescent probe was added by adding the Reagent 1 working solution to the cells. The DCFH-DA working concentration was 20 μM. The solution is now incubated at 37°C for 30 min. After that, we collected the incubated single-cell suspension and centrifuged it at 1,000 g for 10 min to collect cells. These cells were washed with Reagent 3 working solutions two times. Later, we centrifuged the cell suspension and collected the cell precipitation and further resuspended the collected cells with Reagent 3 working solution for detection. Fluoresce intensity was determined at an excitation wavelength of 502 nm and emission wavelength of 525 nm using a fluorescence microplate reader. However, this method has some limitations (Dikalov and Harrison, 2012).

### Reduced Glutathione Assay

Reduced GSH was measured in the mouse mammary adenocarcinoma tissue using a colorimetric method through a reduced glutathione assay kit (Elabscience, United States) catalog no: E-BC-K097-M. A fresh tissue sample was collected and washed with normal saline, the water on the tissue surface was absorbed, and the tissue sample was weighted, and a buffer solution with a protein precipitator was added. 10% homogenate was prepared by mechanical homogenization on an ice bath, centrifuged for 10 min at x10,000 g, and then the supernatant was collected for detection. GSSG is reduced to GSH by glutathione reductase; GSH reacted with DTNB to produce GSSG and TNB yellow color. The amount of yellow TNB was determined by the amount of reduced glutathione. The reduced glutathione was calculated by measuring the optical density value at 412 nm.

### Detection of Cleaved Caspase-3

An immunohistochemistry assay was used to study the cleaved caspase-3 in tumor sections using a conventional avidin-biotin-immunoperoxidase protocol (Elabscience, United States). Tumor samples were fixed in neutral-buffered formalin 10% and processed to prepare paraffin-embedded tissue sections in a standard procedure. Before incubation with the primary antibody (1:50 dilution as supplied by the manufacturer), tissue sections were exposed to heat-induced epitope retrieval by incubation in a water bath with pH 6 and at 98°C (40 min) in a vegetable steamer, followed by cooling at room temperature and treatment with 3% hydrogen peroxide before antibody application and then treatment with rabbit polyclonal anti-cleaved caspase -3 antibody for 30 min at room temperature. Later, samples were washed with phosphate-buffered saline and incubated again with a labeled streptavidin-biotin reagent. Immunoreactive products were visualized with the DAB reaction. The sections were counterstained with hematoxylin for 2 min. The optical density (OD) of cleaved caspase-3 was determined using the FIJI image analysis tool using pictures covering all the sections. The following formula was used to determine the OD values: OD = log(maximum intensity/mean intensity), with a maximum intensity equal to 255 (Mustafa et al., 2015).

### Statistical Analysis

All data analyses were performed with Graph Pad Prism version 8.01 (GraphPad software. CA, United States) and Excel version 10. Data were analyzed using one-way ANOVA analysis, which was used to perform comparison between groups. All data were presented as mean and standard deviation. The significance level was set at *p 0.05, **p 0.01, and ***p 0.001.

## Result

### Acarbose Markedly Enhances the Antitumor Efficacy of Newcastle Disease Virus

**FIGURE 1:**
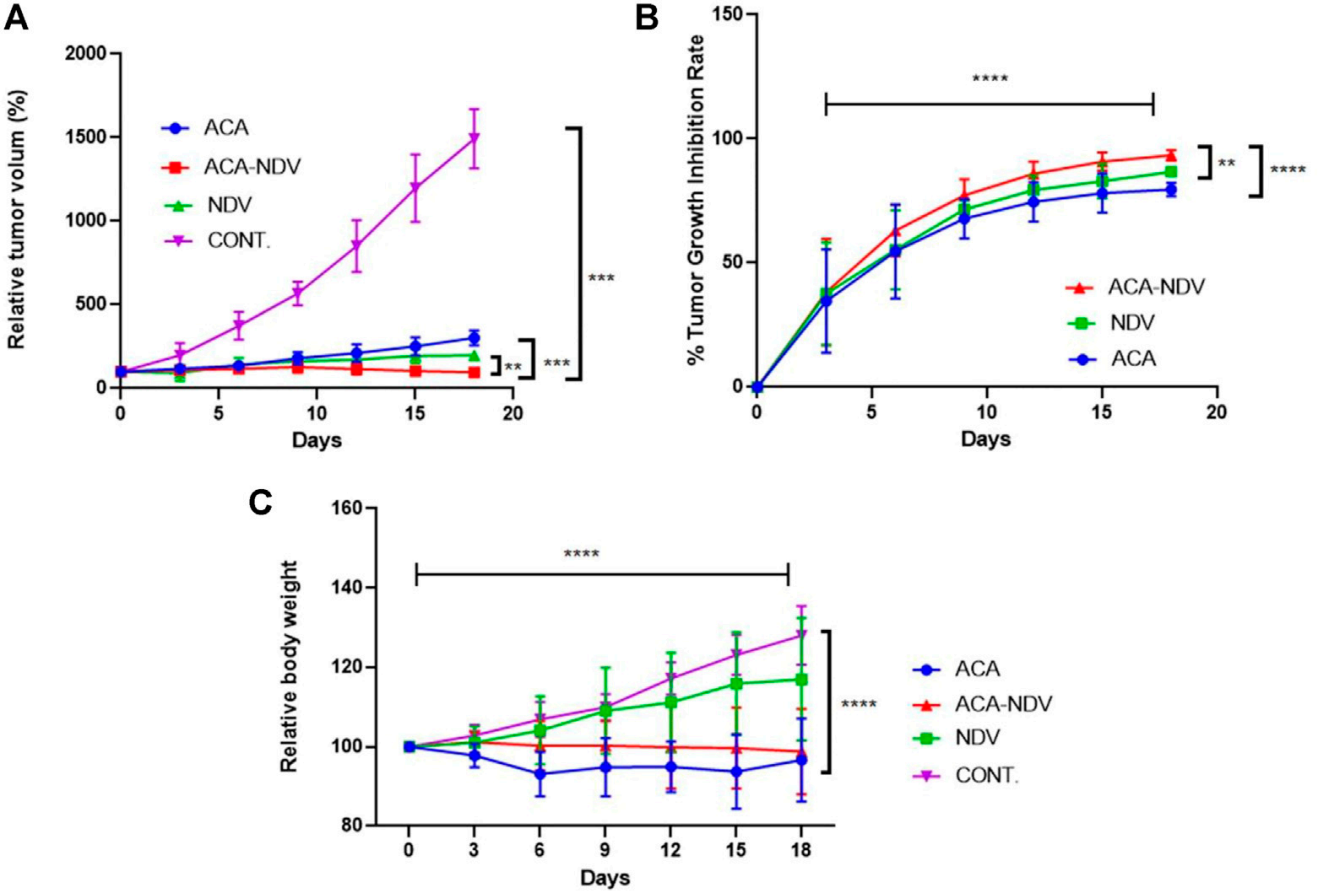
Antitumor efficacy of acarbose (ACA), Newcastle Disease Virus (NDV), and a combination of both against a mammary adenocarcinoma AN3 *in vivo* model. **(A)** Relative tumor volumes over 18 days were plotted. Compared to the control group, ACA, NDV, or a combination of both generated a significant (*p* < 0.0001) decrease in relative tumor volume. Compared to both monotherapy groups, the combination therapy of acarbose and Newcastle Disease Virus (ACA-NDV) demonstrated significantly greater tumor size reduction. **(B)**e Growth inhibition curve demonstrated that the combined therapy group had the highest overall tumor growth inhibition, followed by the NDV group. The ACA group showed the least growth inhibition. In the control group, the tumors continued to grow during the experiment. **(C)** ACA efficiently reduced body weight in mice bearing breast cancer. Bodyweight measured after treatment with ACA, NDV, and combination every 3 days indicates that ACA induces a marked decrease in body weight. By contrast, NDV did not induce a significant effect on body weight. ∗∗∗∗ *p* < 0.0001, ∗∗∗ *p* < 0.001, ∗∗ *p* < 0.01, and ∗ *p* < 0.05versus CONT.

We conducted an *in vivo* experiment to examine the efficacy of the combined treatment of ACA-NDV compared to monotherapies on tumor volume of the mouse breast cancer model. Relative tumor volume was plotted over an 18-day treatment period, as shown in Figure 1A. All treatment modalities resulted in a statistically significant (*p* < 0.0001) decrease in tumor volume compared to the untreated control group. The ACA–NDV combined treatment significantly reduced tumor size (*p* < 0.0001) compared to the ACA and NDV mono treatment groups. Additionally, the combination therapy group achieved the highest tumor growth inhibition rate (93.34%), followed by the NDV group (86.75%). As shown in Figure1B, the lowest growth inhibition rate was found in the ACA group (79.64%). Tumors continued to grow in the untreated control group throughout the experiment.

### Combined Acarbose-Newcastle Disease Virus Treatment Efficiently Maintains Mouse Body Weight Compared to Acarbose Alone

The study aimed to explore whether ACA, NDV alone, or a combination reduced body weight in mice bearing breast cancer. Combined ACA-NDV treatment efficiently maintains mouse body weight compared to ACA alone, while ACA treatment resulted in a noticeable decline in body weight (Figure 1C). By contrast, NDV alone did not affect body weight.

### Acarbose and Combined Acarbose-Newcastle Disease Virus Treatments Induce Glucose Deprivation

**FIGURE 2:**
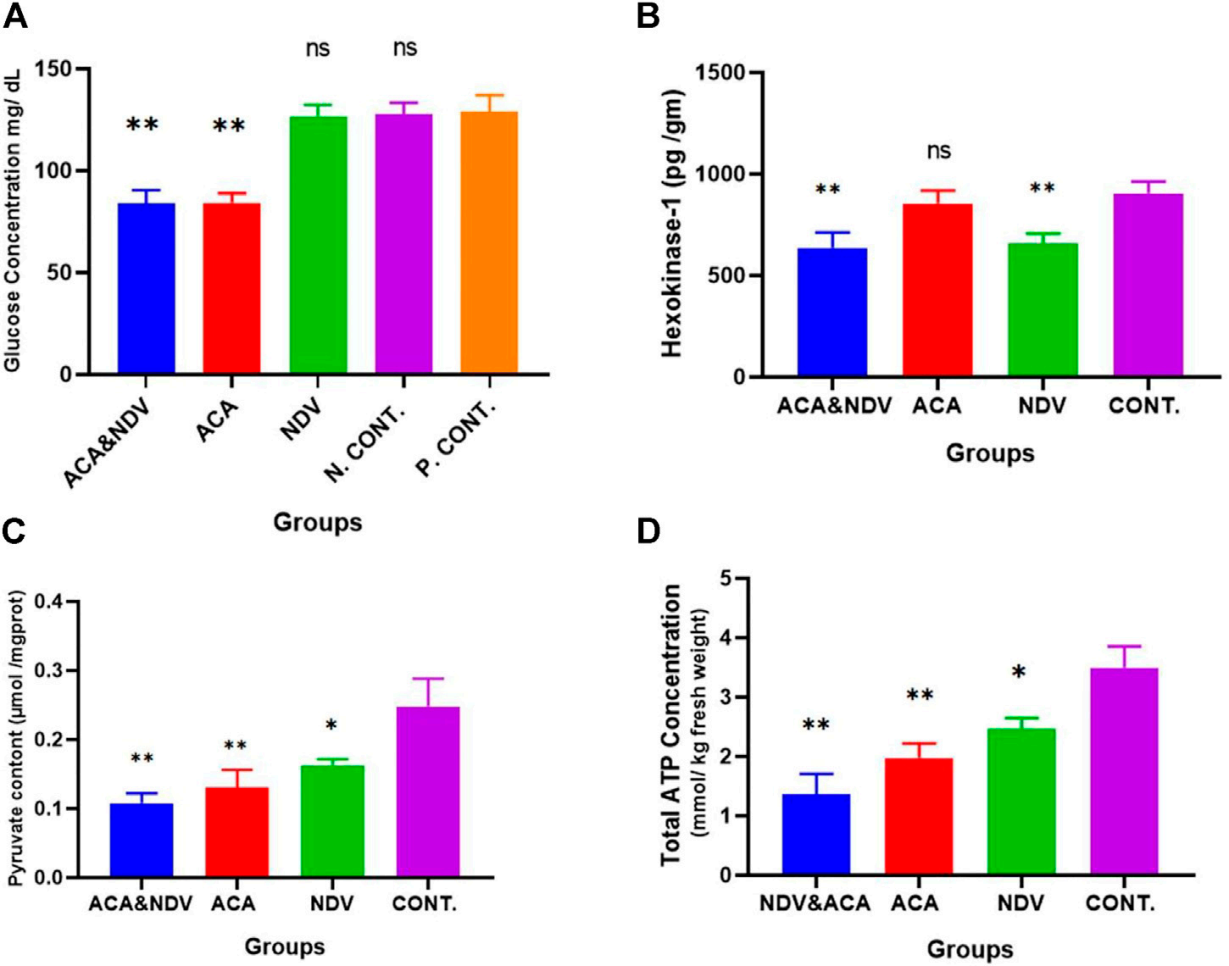
ACA induces glucose deprivation. **(A)**, Glucose measured after Acarbose (ACA) treatment, Newcastle Disease Virus (NDV), and a combination of Acarbose and Newcastle Disease Virus (ACA-NDV), ACA exhibited a significant ∗∗ *p* < 0.01 decreased level of glucose in ACA and ACA-NDV groups compared with the positive control (P. CONT) (bearing breast cancer) and negative control N. CONT. (healthy mice) groups. **(B)** ELISA assay quantified the concentration of the Hexokinase enzyme. We observed a significant decrease in the expression of the HK-1 protein in the NDV- and ACA-NDV-treated group compared to the untreated control group. ACA alone had no significant impact on HK-1 concentration. **(C)** Measurement of pyruvate content. **(D)** Measurement of total ATP concentration. In combination with NDV, ACA efficiently inhibits glycolysis product (pyruvate) and ATP. To confirm the effect of ACA and NDV combination therapy on glycolysis products, we examined pyruvate and total ATP level in tumor tissue. We found that the ACA-NDV treatment had significantly reduced glycolysis product (pyruvate) and ATP compared to the untreated group. ∗∗∗ *p* < 0.01, ∗∗ *p* < 0.01, and ∗ *p* < 0.05 versus Cont.

We next sought to determine whether there is a relationship between ACA, NDV, and its combination with glucose deprivation. We detected glucose concentration in the blood to confirm the effect of ACA and NDV on the glucose level. As expected, compared with the control group, ACA exhibited a decreased level of glucose (Figure 2A). Furthermore, combined ACA-NDV treatment exhibited the same level of glucose level reduction. NDV alone does not affect glucose levels in the blood.

### Newcastle Disease Virus-Acarbose Combined Treatment Efficiently Decreases Hexokinase Enzyme Level in Tumor Tissue

The present study identified and quantified HK-1 enzyme expression in the tumor tissue after treatment. The ELISA assay was used to assess the enzyme quantity according to the manufacturer’s protocol. The HK enzyme level was compared between treated and untreated groups (Figure 2B). We detected a significant drop in the expression of the HK-1 enzyme in the (NDV and ACA–NDV) treated groups compared to the untreated control group (659.88, 636.09, and 904.22 pg/ml). The results indicated that the combination of ACA-NDV considerably decreased the HK-1 enzyme concentration. ACA alone had no noticeable effect on the concentration of HK-1. These findings imply that NDV may play a critical role in suppressing glycolysis metabolism in cancer.

### Acarbose Suppresses Glycolysis Product (Pyruvate) and Total ATP Effectively in Breast Cancer When Combined With Newcastle Disease Virus

The impact of ACA-NDV combination therapy on glycolysis products was investigated. We examined the pyruvate and total ATP level in tumor tissue. We observed that ACA-NDV efficiently decreased glycolysis product (pyruvate Figure 2C and ATP Figure 2D) compared to the control group and better than monotherapy modalities.

### Acarbose-Newcastle Disease Virus Combination Treatment Induces Oxidative Stress

**FIGURE 3:**
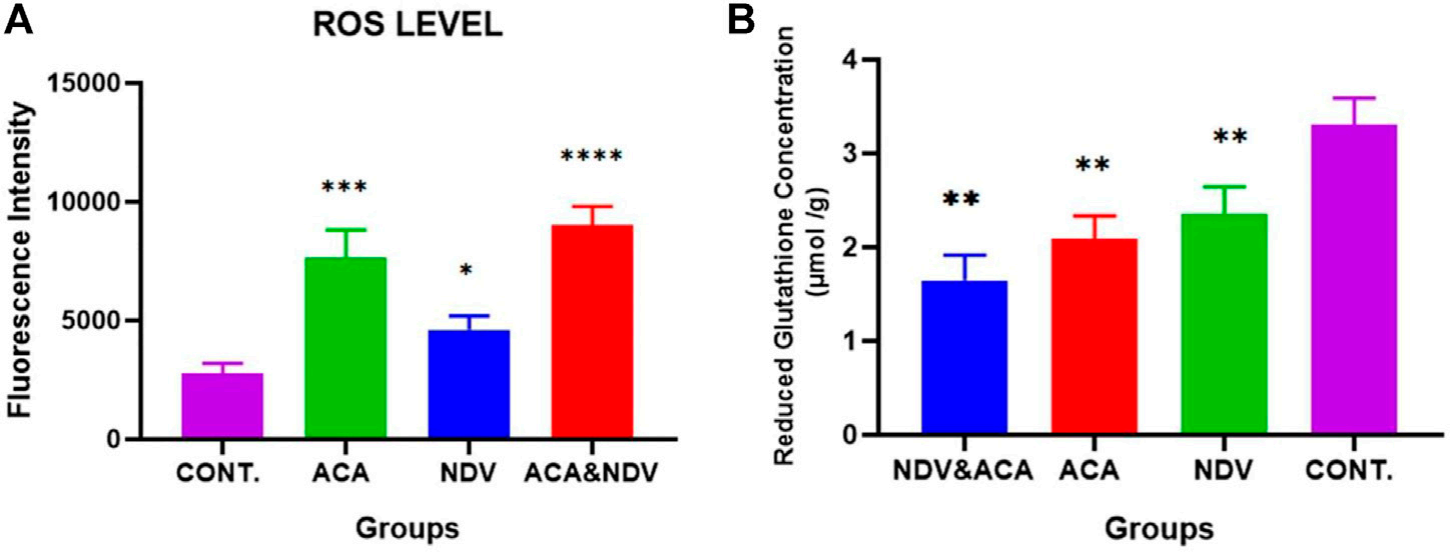
ACA and NDV combination induces metabolic oxidative stress. We detected ROS and reduced GSH in breast cancer tissue, and we observed that ROS levels were significantly increased in treated groups (ACA, NDV, and ACA-NDV) than in the untreated groups. Reduced GSH levels were lower in ACA-NDV combination therapy than in the untreated group. **(A)** Measurement of ROS level. **(B)** Measurement of reduced glutathione concentration. ∗∗∗∗ *p* < 0.0001, ∗∗∗ *p* < 0.001, ∗∗ *p* < 0.01, and ∗ *p* < 0.05 versus CONT.

To investigate whether ACA-NDV induced oxidative stress in tumor tissue, we measured ROS and reduced GSH in breast cancer tissue. We observed high ROS levels in the treated groups than in the control untreated group, but ACA-NDV combined therapy induced higher levels of ROS than monotherapies (Figure 3A). Reduced GSH levels were lower in ACA-NDV combination therapy than in other treated and untreated groups (Figure 3B).

### Acarbose-Newcastle Disease Virus Combination Therapy Induces Apoptosis

**FIGURE 4:**
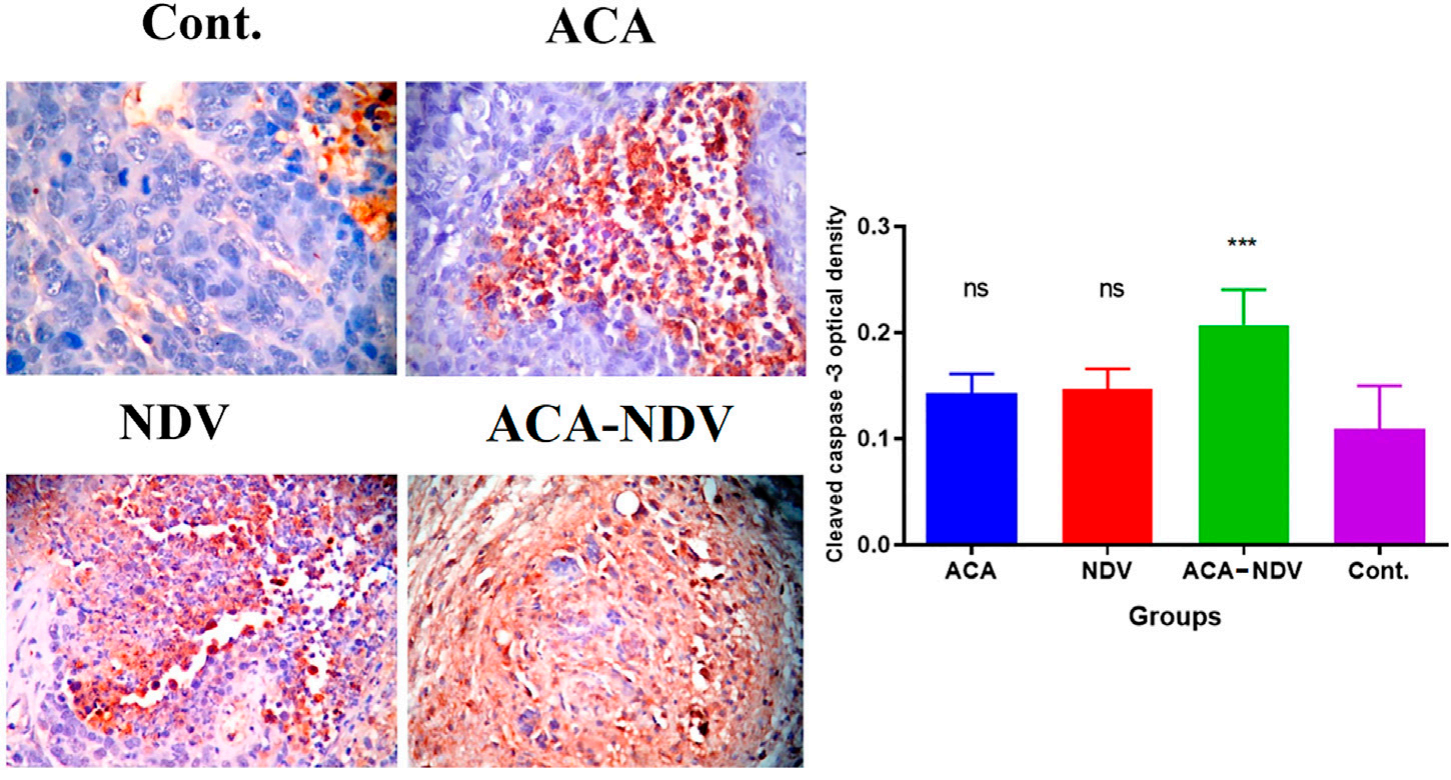
ACA-NDV combination therapy induces apoptosis, confirmed by immunohistochemistry analysis for cleaved caspase-3. Results showed a mild effect on breast cancer cells in monotherapy (ACA and NDV alone)-treated groups, while the combination therapy of ACA-NDV has more cas-3 expression than the single therapy. ∗∗∗ *p* < 0.001 versus Cont.

The efficacy of ACA-NDV to induce apoptosis in breast cancer tissue was proven by immunohistochemistry analysis for the cleaved caspase-3 levels of expression. Combination therapy of ACA-NDV induced higher expression levels for cleaved caspase-3 than single therapy and untreated control groups. Single-therapy groups had no significant cleaved caspase-3 expression levels compared with the untreated control group (Figure 4).

## Discussion

The findings of this study demonstrate that a combination of glucose deprivation (using ACA) and virotherapy using oncolytic NDV can synergistically suppress breast cancer growth and induce breast cancer cell apoptosis *in vivo*. The combination of ACA and NDV was more effective at inhibiting the glycolysis pathway and inducing oxidative stress than each treatment administered alone. Breast cancer is associated with malignant tumors that lead to poor prognosis in women (Soerjomataram et al., 2008). Chemotherapy and radiotherapy for breast cancer have limited efficacy (Hickey et al., 2013). Cancer cells possess enhanced glycolysis and reduced oxidative phosphorylation (OXPHOS) capacity (Zheng, 2012). Aerobic glycolysis is preferred because it generates less ROS than mitochondria (Cairns et al., 2011). The increase in the uptake of glucose by cancer cells for use as a carbon source for anabolic processes, including nucleotides, proteins, and lipids, is needed to support cell proliferation (Liberti and Locasale, 2016). Cancer cells generate energy primarily by increasing the rate of glycolysis by 200 times than that of normal cells of origin (Alfarouk et al., 2014). In the presence of extracellular glucose and robust glucose transport, glycolysis drives more rapid ATP production (albeit less efficient) than ATP production *via* mitochondrial oxidative phosphorylation. Aerobic glycolysis also benefits cancer cells because it generates less ROS and allows the cells to adapt to the intermittently hypoxic conditions prevalent in a poorly vascularized tumor. The decreased glucose concentration in the cancer cell leads to pushing and activating mitochondrial oxidative phosphorylation, which causes increased ROS because of the defect in mitochondria. At the same time, it decreases ATP production because of reduced pyruvate levels due to glucose deprivation (Cairns et al., 2011). Recently it was discovered that glucose deprivation induced oxidative stress and cytotoxicity in cancer cells (Ahmad et al., 2005).

This study showed a reduction in blood glucose concentration in ACA-treated groups compared with the untreated control groups. Our results confirm the findings of an earlier study that ACA causes a reduction in glucose levels (Mustafa et al., 2015), resulting in induced glucose deprivation. Moreover, ACA (glucose deprivation inducer) reduced relative body weight compared with the control group. ACA inhibits alpha-amylase and alpha-glucosidase; therefore, delayed absorption of complex carbohydrates from the intestine leads to decreased glucose level and body weight (Zhang et al., 2020).

We measured relative tumor volume and tumor growth inhibition to determine whether free ACA or combination with NDV has an antitumor effect. We found that the ACA-NDV combination significantly reduced tumor volume compared with the untreated group. This combination had higher tumor growth inhibition than monotherapies. The Iraqi NDV AMHA1 strain recently showed anticancer properties through glycolysis pathway inhibition and apoptosis induction (Al-Ziaydi et al., 2020a). In addition to glycolysis inhibition, NDV has several antitumor mechanisms. One of these mechanisms activates the immune system by inducing cytokine secretion (IL-2 and IFN-gamma) and attracts CD56 natural killer and CD8 cytotoxic lymphocytes into infected cancer tissue (Washburn and Schirrmacher, 2002; Al-Shammari et al., 2011). Moreover, NDV is replicated within the AN3 tumor mass for many cycles after intratumoral injection, which leads to activation of caspase-3 in cancer cells (Hickey et al., 2013); therefore, this mechanism reduces tumor volume and enhances the antitumor efficacy of NDV. The current study demonstrates that ACA decreases tumor growth, and the antitumor effect may be due to ACA-induced glucose deprivation, which leads to increased ROS formation and creates oxidative stress that activates apoptosis (Graham et al., 2012).

HK1 has a key role in the glycolysis pathway at the first step *via* converting glucose to glucose-6-phosphate. Previous studies reported that the glycolysis-related gene (HKI) was overexpressed and participated in tumorigenesis; it acts as a poor prognosis biomarker in many cancers (He et al., 2016; Dai et al., 2020). Thus, we conducted HK-1 quantification by ELIZA assay. The result showed that ACA had a nonsignificant effect on HK-1 levels, which may be because ACA is an alpha-glucosidase inhibitor and acts majorly in the intestine (Kalra, 2014). Nevertheless, groups infected with NDV revealed a reduction in HK-1 levels. Our result is similar to the previous report confirming that NDV inhibits the activity of HK (Al-Ziaydi et al., 2020b). It has been reported that NDV may downregulate other glycolysis-related enzymes, such as fructose-bisphosphate aldolase C (ALDOC) and phosphoglycerate kinase (PGK). This downregulation is explained as an alteration in protein expression during the NDV infection process, which may support the control host responses to virus invasion through cell signaling pathways controlling to regulate the infection course (Deng et al., 2014).

To further confirm that this combination of ACA-NDV inhibits the glycolysis pathway in breast cancer cells, we measured products of glycolytic pathway levels (pyruvate and ATP) in breast cancer tissue. The findings revealed that the ACA-NDV combination treatment suppresses pyruvate and ATP compared with the monotreatments and the untreated group. Depending on the glucose result, ACA decreases glucose concentration, resulting in reduced pyruvate concentration in groups treated with ACA (Walton et al., 1979). Pyruvate-level reduction in ACA-NDV may be due to this combination of decreased HK activity and diminishing pyruvate concentration (Al-Ziaydi et al., 2020a). The inhibition of glycolysis causes a decline in pyruvate formation and thus a depletion of ATP (Al-Ziaydi et al., 2020b).

In support of our hypothesis, we determined intracellular ROS formation results in ACA treatment (glucose deprivation inducer) and infection with NDV in breast cancer tissue. We found that intracellular ROS formation increased in the combination ACA-NDV group compared with the control group. ACA alone and NDV alone also increased ROS levels to lesser degrees than combined ACA-NDV treatment.

Our results suggest that glucose deprivation induced by ACA and oncolytic NDV can activate a positive response loop, including intracellular ROS generation by mitochondria and NADPH oxidase, which was described individually by others (Ahmad et al., 2005; Keshavarz et al., 2020). In addition, our result of ACA-NDV treatment induced oxidative stress, which led to reduced GSH depletion in the treated groups.

Pyruvate and NADPH have involved glucose metabolism products from glycolysis and pentose cycle; this product functions as anti-hydroperoxide. Pyruvate removed ROS through a direct reaction with hydrogen peroxide; this causes the decarboxylation of pyruvate to produce acetic acid and converts H_2_O_2_ to H_2_O (Nath et al., 1995). In addition, NADPH was utilized as a cofactor for glutathione reductase to reduce glutathione disulfide and then detoxify ROOH and H_2_O_2_ by glutathione peroxidases (Sies et al., 2017). Therefore, the increased uptake of glucose by cancer cells is necessary to overcome increased intracellular ROS generated from metabolic-, genetic-, and microenvironment-associated alterations in cancer cells (Ghanbari Movahed et al., 2019). In correlation with this mechanism, we noticed that the ACA-NDV combination diminished reduced GSH. A decline in the pentose phosphate pathway accompanies glucose deprivation, dysfunction of glutathione synthesis and ROS accumulation, and a decrease in the NADPH and intracellular GSH (Zhu et al., 2020). The present study finding is consistent with previous works that reported NDV inhibition of glutathione synthesis, underexpression of glutathione peroxidase, and accumulation of ROS in tumor cells (Kan et al., 2021; Obaid et al., 2021).

Cancer cells escape the apoptotic pathway through various means, including mitochondrial pathway impairment, underexpression of pro-apoptotic proteins, and overexpression of anti-apoptotic proteins (Kluck et al., 1997; Pfeffer and Singh, 2018). Therefore, ACA (glucose deprivation inducer) and NDV synergize to overcome cancer resistance to apoptosis. In the current study, cleaved caspase-3 detection using immunohistochemistry showed that the ACA-NDV combination was the best inducer for apoptosis compared with ACA alone or NDV alone. Previous works reported that NDV induces apoptosis in caspase-dependent, caspase-independent, and endoplasmic reticulum pathways (Al-Shammari et al., 2012; Mohammed et al., 2019). A recent report postulated that NDV induces ferroptosis in tumor cells exposed to nutrient deprivation (Kan et al., 2021). Moreover, ACA-induced glucose deprivation displays cleavage of caspase and caspase substrates, which induces apoptosis (Caro-Maldonado et al., 2010; Obaid et al., 2022). Also, GD-induced stress promotes both TRAIL-RD/DR2 and receptor-mediated apoptosis (Iurlaro et al., 2017). In addition, glucose deprivation induces inhibition of glycolysis, leading to lack of proton provision and mitochondrial electron transfer chain constant proton consumption to generate energy. This deficiency in the proton is compensated by lysosomes through proton efflux, leading to an increase in lysosomal pH, resulting in necrosis or apoptosis depending on alkalinization extent (Cui et al., 2017).

In conclusion, this study’s results strongly support the novel hypothesis that ACA induces glucose deprivation with virotherapy synergizing to promote metabolic oxidative stress and apoptosis. This study is the first to report that ACA-induced glucose deprivation synergizes with oncolytic NDV, featuring a very smart glycolysis pathway targeting safe and effective therapy. This novel combined therapy has a strong translational capacity in clinical therapy.

## Data Availability

The raw data supporting the conclusions of this article will be made available by the authors, without undue reservation.
